# Characterization of flue gas desulphurized (FGD) gypsum of a coal-fired plant and its relevant risk of associated potential toxic elements in sodic soil reclamation

**DOI:** 10.1038/s41598-023-45706-y

**Published:** 2023-11-13

**Authors:** Parul Sundha, Raj Mukhopadhyay, Nirmalendu Basak, Arvind Kumar Rai, Sandeep Bedwal, Subedar Patel, Sanjay Kumar, Harshpreet Kaur, Priyanka Chandra, Parbodh Chander Sharma, Sanjeev Kumar Saxena, Somendra Singh Parihar, Rajender Kumar Yadav

**Affiliations:** 1 ICAR–Central Soil Salinity Research Institute, Karnal, Haryana 132 001 India; 2 National Thermal Power Corporation, Vindhyachal, Singrauli, Madhya Pradesh India

**Keywords:** Environmental sciences, Environmental chemistry, Environmental impact

## Abstract

Thermal Power Plant generates FGD gypsum as by-product during coal combustion. This study evaluates the characterization (spectroscopic and elemental), potentially toxic elements (PTEs) distribution, and environmental risk assessment of FGD gypsum for safe and sustainable use in agriculture. The XRD and SEM analysis confirmed the dominance of crystalline CaSO_4_·2H_2_O in FGD gypsum. The order of concentrations of PTEs in FGD gypsum was Fe > Al > Mn > Zn > Ni > Co. The residual fraction was the dominant pool, sharing 80–90% of the total PTEs. The heavy metals (HMs) were below the toxic range in the leachates. The Co, Ni, Al, Fe Mn, Zn had low (< 10%) risk assessment code and the ecotoxicity was in the range of 0.0–7.46%. The contamination factor was also low (0.0–0.16) at the normal recommended doses of FGD gypsum application for sodicity reclamation. The enrichment factor was in the order of Al < Mn < Co < Zn < Ni. Mn [enrichment factor (E_f_) 1.2–2.0] and Co (E_f_ 1.7–2.8) showed negligible enrichment of metals, whereas Ni (E_f_ 4.3–5.2) and Zn (E_f_ 4.5–5.6) reported moderate accumulation in soil. The application of FGD gypsum @ 10 t ha^−1^ for sodicity reclamation will develop a geo-accumulation index below the critical values indicating its safe and sustainable use to achieve land degradation neutrality (LDN) and UN’s Sustainable Development Goals.

## Introduction

Coal is one of the major sources of energy for power generation in the world^1,2^. The burning of coal releases many harmful gases (SO_x_, NO_x_), particulate matter, and heavy metals (HMs) into the environment which adversely affect human health^3,4^. Therefore, most power plants have upgraded air pollutant devices to capture the most obnoxious gases released from thermal power plants worldwide. The Ministry of Environment, Forest, and Climate Change (MoEFCC, 2015) of India fixed the limits of emitting such obnoxious gases from thermal plants which forced them to upgrade their electrostatic precipitators, installation of flue gas desulphurized (FGD) system and fine-tuning of boiler operations^5^. FGD system limits the escape of SO_x_ from flue gas by spraying the wet limestone, which reacts with the SO_x_ in the flue gas producing calcium sulphate di-hydrate [CaSO_4_·2H_2_O] by-product, known as FGD gypsum^6^. Countries like the United States, China, and Germany, have preferably adopted the wet system of FGD for handling the flue gasses^7^.

The availability and quality of the mined mineral gypsum is one major concern for agricultural soils. India imported around 80% of gypsum to fulfill the consumption of around ten million tonnes (Mt) of gypsum in the year 2014–15^5,8^. The FGD gypsum could be an alternate option for the management of salt-affected soils across the world accounting for 99.6% of CaSO_4_·2H_2_O (24.3% of Ca and 18.5% of S content)^8–11^. The estimated production of FGD gypsum from India’s power plants remains around 12–17 Mt per annum to meet the national shortfall and shrink the import load of gypsum. Moreover, India has 6.73 million hectares (Mha) of salt-affected land, out of which 3.77 Mha is sodic soil which can be reclaimed by the application of mineral gypsum or other alternates like FGD gypsum^12–14^. Gypsum on dissolution supplies Ca^2+^_sol_, which neutralizes soil alkalinity (NaHCO_3_/NaHCO_3_), and some of the Ca^2+^_sol_ replaces Na^+^ of the clay micelles^14^ (Eqs. 1 and 2).

1 $${\text{NaHCO}}_{{3}} /{\text{NaHCO}}_{{3}} + {\text{Ca}}^{{{2} + }} + {\text{ SO}}_{4}^{2 - } = {\text{Na}}_{{2}} {\text{SO}}_{{4}} \left( {{\text{leachable}}} \right) \downarrow + {\text{CO}}_{{2}}$$

2 $${\text{Na}}^{ + } {-}\left[ {{\text{Clay}}\;{\text{micelle}}} \right]{-}{\text{Na}}^{ + } + {\text{Ca}}^{{{2} + }} + {\text{ SO}}_{4}^{2 - } \left[ {{\text{Clay}}\;{\text{micelle}}} \right]{-}{\text{Ca}} + {\text{Na}}_{{2}} {\text{SO}}_{{4}} \downarrow \left( {{\text{leachable}}} \right)$$

Coal contains many of the trace HMs such as Cr, Cd, Ni, As, Pb, Hg, and Se that exist in the natural environment^15^, and accumulate into different coal by-products during the combustion and pollution control processes^16^. The trace elements present in the coal are classified into three groups, *i.e*., non-volatile elements (Rare earth elements, Ca, Fe, Al, Si, Hf, Th, Zr, etc.), volatile (Cu, Zn, As, Cd, Pb, Mo, etc.), and very volatile (B, N, S, Hg, Se, halogens)^17,18^. Therefore, the elemental, surface, and mineral characterization of FGD gypsum, chemical speciation/fractions of HMs (ion-exchangeable, bound to carbonate, Fe–Mn oxides, sulfides, and organics and residual) in FGD gypsum is essential to understand the presence of toxic elements, HMs fraction and phase in FGD gypsum, and its environmental risk when used as an alternative to mineral gypsum for reclamation and recycling purposes^8,19^.

**Figure 1:**
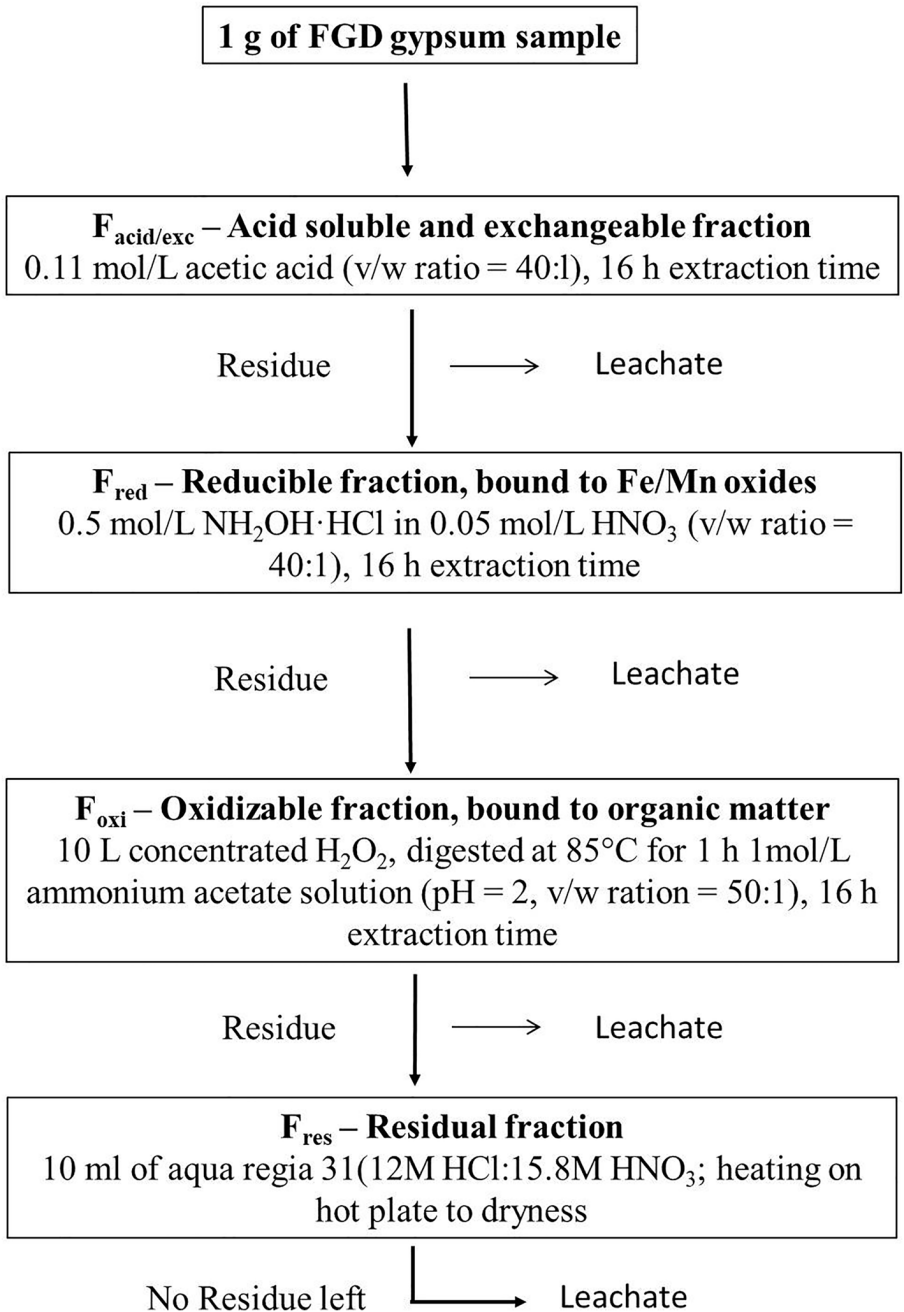
The flow diagram of selective sequential extraction (SSE) procedure^36^.

The study of mineral characterization, the thermal stability of elements, and the chemical speciation of HMs present in FGD gypsum helps to understand their concentration, mobility, and bioavailability in FGD gypsum. Research has been done on the partitioning behaviour and the chemical speciation of the metals in the by-products of the desulfurization process^20^. The FGD gypsum of Shanxi province of China contained 77.4% of Mn, 25% of total Pb, and 51.8% of Zn as easily-soluble forms showing higher mobility^21^. Further, the selective sequential extraction method (SSEM) analyzed the chemical speciation of trace elements in FGD gypsum and found the bioavailability of metals decreased following the order: Mn > Zn > Cd > Cr > Pb > Ni, while the mobility decreased in the order: Cd > Mn > Ni > Pb > Zn > Cr. The study of mineral characterization and leaching toxicity of Hg in FGD gypsum samples collected from seventy power plants in twenty provinces of China indicated the complex behaviour of Hg with lesser metal mobility^22^. Another trace metal, arsenic (As) present in the FGD residues from Pennsylvania power plants showed a strong association with Fe–Mn oxides^23^ occurring mainly as a residual form (50.1–73.7%) in FGD gypsum of China^24^. Other factors affecting the leaching characteristics include pH, solid solution ratio, and leaching time^24–26^. The ecological risk of the HMs present in the FGD gypsum is better understood through the estimation of pollution indices^27,28^. These indices will serve as a tool to assess the ecotoxicological pollution through contamination, enrichment, and accumulation of metals in soil under short or long-term application of FGD gypsum in agricultural lands^24^. As a huge amount of FGD gypsum is produced in thermal plants of India every year, therefore, research should be concentrated on the distribution, speciation or fractionation, leaching toxicity, and ecotoxicological risk assessment of other HMs along with elemental, surface, and mineral characterization of FGD gypsum. Apart from HMs like Hg, As, the information on chemical speciation/fractionation, and leaching toxicity of other HMs is highly lacking in the literature, particularly in India’s perspective which should be taken into consideration to understand the environmental risks of FGD gypsum, provide guidelines of treatment, disposal and application rate as amendment of the FGD by-products and minimize the environmental pollution in India. Therefore, the present study was formulated to (1) characterize the mineral and elemental composition of FGD gypsum received periodically from the coal plant of National Thermal Power Corporation (NTPC), Vindhyachal, Singrauli, Madhya Pradesh, India; (2) evaluate the risk assessment and eco-toxicological risk of HMs present in FGD gypsum to use it as an alternative to mineral gypsum for reclamation of degraded sodic soil to achieve land degradation neutrality (LDN) and United Nation’s Sustainable Development Goals (SDGs) in India (Fig. 1).

## Results and discussion

### XRD, SEM and elemental characterization of FGD gypsum

**Figure 2:**
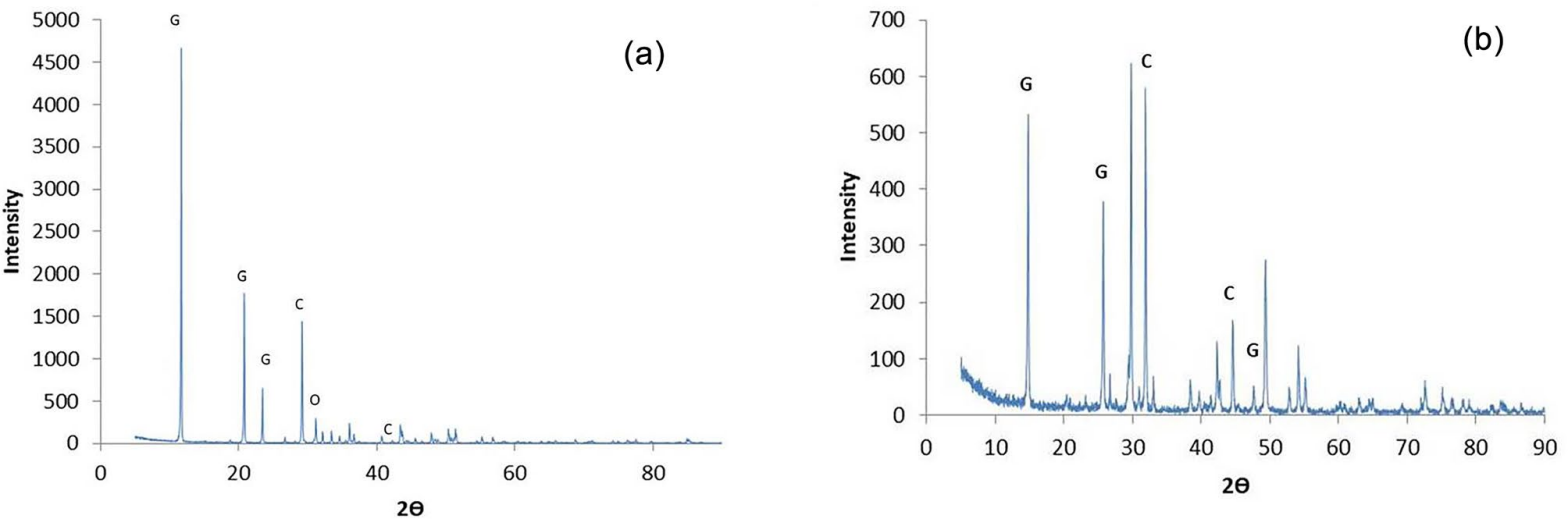
XRD analysis of (**a**) FGD gypsum 1, (**b**) FGD gypsum 2; G: Gypsum (CaSO_4_. 2H_2_O); C: Calcite (CaCO_3_), Q: Quartz (SiO_2_).

X-ray diffraction analysis revealed that the characteristic peaks at 2θ value of 11.8, 20.8 and 23.5 (Fig. 2a) of the FGD gypsum 1 suggested that crystalline (CaSO_4_·2H_2_O) gypsum was the dominant mineral present in the FGD gypsum by-product. Some traces of quartz were also observed from the peaks at 2θ value of 31.2. Fu et al.^18^ also mentioned the presence of quartz in FGD gypsum produced from the FGD system of a coal power plant in China. Traces of calcite were also observed in FGD gypsum 1 at 2θ value of 29.2 and 42.2. Similarly, for FGD gypsum 2 the samples showed the characteristics peaks of gypsum at 2θ value of 14.8, 25.7, and 49.3 (Fig. 2b). Few traces of calcite appeared with characteristic peaks of 2θ value of 29.8 and 42.3, while the traces of quartz were observed at characteristics peak at 2θ value of 31.9 and 44.6. Similar characteristics peaks of gypsum, quartz, and calcite in FGD gypsum by-products were also reported by Fu et al.^18^, Hao et al.^21^. The traces of quartz and calcite present were the impurities incorporated during FGD gypsum generation from the coal plant. The source of these impurities could be the raw coal used or the limestone used in the desulfurization process. However, a higher amount of quartz and calcite will interfere with the functioning of FGD gypsum in sodic soil reclamation process^29,30^.

**Figure 3:**
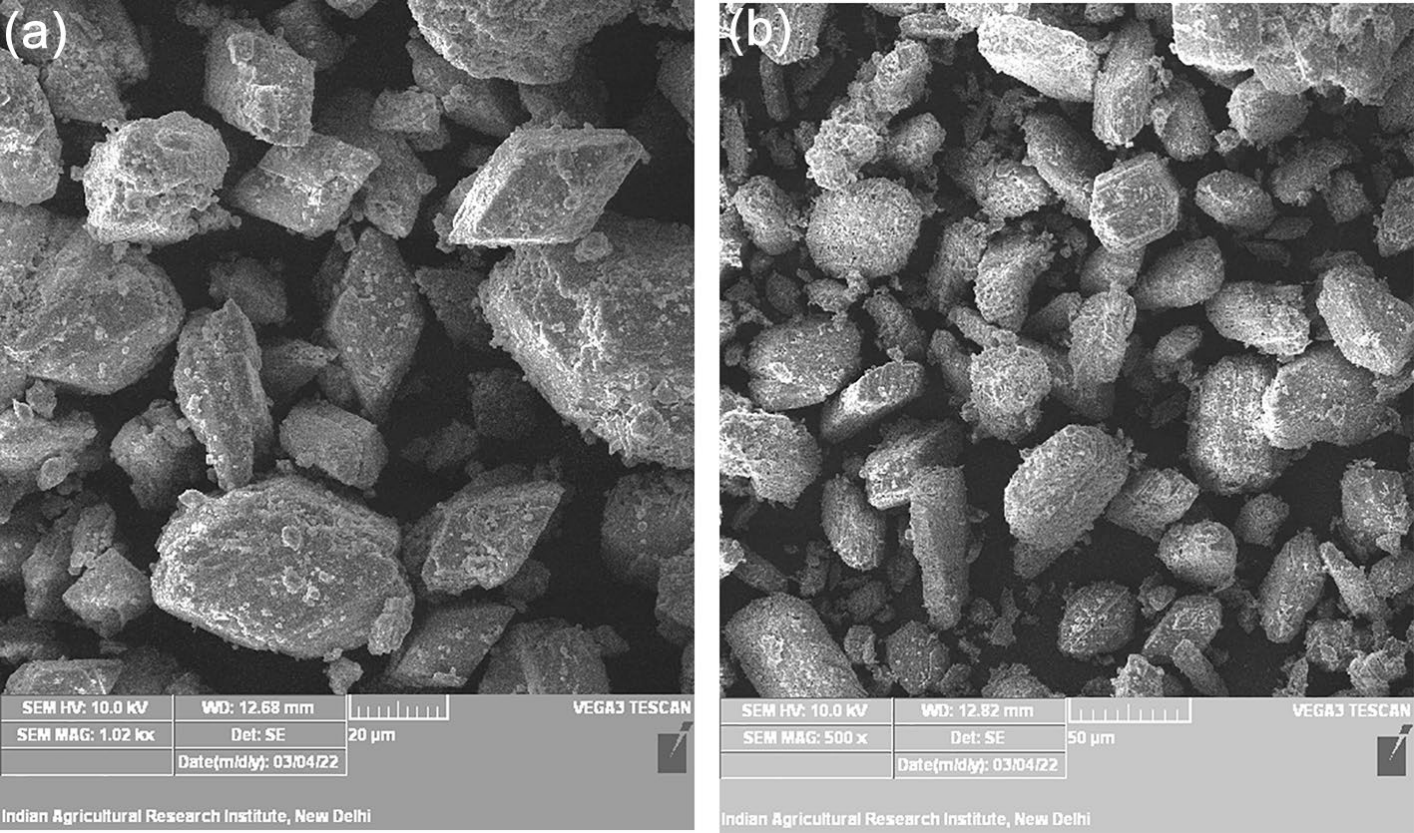
SEM images of (**a**) FGD gypsum 1, (**b**) FGD gypsum 2.

The SEM images of FGD gypsum 1 (Fig. 3a) and FGD gypsum 2 (Fig. 3b) showed the presence of a clear flaky crystal-like structure of gypsum in both the FGD gypsums^18,25^. Ca and S are the dominant elements found in FGD gypsums with concentrations of 276.2 and 186.5 g kg^−1^ and other trace elements like Si and Mg with concentrations of 1.94 and 8.7 g kg^−1^ were observed in elemental analysis of FGD gypsum (Table 1). Elemental analysis of FGD gypsum performed by Fu et al.^18^, Li et al. (2015)^31^ also indicated the dominance of Ca and S and the presence of Si and Mg as trace (Table 1). Since, there was no such significant difference between the three FGD gypsum samples in surface morphology through SEM, and diffraction angle and peaks through XRD analysis, therefore, XRD and SEM analysis were conducted only for two samples i.e. FGD gypsum 1 and FGD gypsum 2. The FGD gypsum samples had an average pH value of 8.73 and EC 4.87 dS m^−1^. The calcium carbonate percentage ranged from 15 to 17% for different FGD gypsum samples with a moisture content of 16.41% (Table 1).

**Table 1:** Characteristics of FGD gypsum samples (mean ± standard deviation; n = 9).

Parameter	Unit	Range	Mean
pH_1:2_		8.69–8.78	8.73 ± 0.03
EC_1:2_	dS m^−1^	4.51–96	4.87 ± 0.14
Moisture content	%	15–17	16.41 ± 0.87
CaCO_3_	%	1.5–5.0	3.12 ± 1.62
Total calcium	g kg^−1^	263.5–287.4	276.16 ± 8.1
Total magnasium		8.0–9.9	8.69.14 ± 0.6
Total silicon		1.20–1.93	1.54 ± 0.0.25
Total sulphur		174.6–204.6	186.50.6 ± 10.2
Total sodium	mg kg^−1^	110.06–135.25	125.13 ± 7.7
Total potassium		597.72–816.53	722.84 ± 69.1
Total phosphorous		273.33–445.02	385.41 ± 55.2

### Total concentration of HMs in FGD gypsum

The concentration of twenty-five potential elements was analyzed. Out of these Al, Co, Fe, Mn, Ni, Zn, Mg, S, NA, K, P, Ca were detected in different samples. As, B, Ba, Cd, Cr, Cu, Hg, Li, Mo, Pb, S, Sb, and V were not detected through ICP-OES estimation. The total concentration of detected PTEs was in the ranges of 1.4–1.7 mg kg^−1^, 7.0–7.2 mg kg^−1^ and 23.4–24.7 mg kg^−1^ for Co, Ni, and Zn, respectively (Table 2). The total concentrations of Mn, Fe, and Al varied from 68.2 to 76.9 mg kg^−1^, 2456.5 to 2697.1 mg kg^−1^ and 1489.4 to 1863.5 mg kg^−1^, respectively. Among the three FGD gypsum samples, only Al in FGD gypsum 3 was significantly (*p* < 0.05) greater than FGD gypsum 1 and FGD gypsum 2. The study of metals in FGD gypsum from different coal-fired power plants have been extensively done in China^18,28,32^.

**Table 2:** Distribution of heavy metals (mg kg^−1^) in different fractions of FGD gypsum according to the sequential extraction procedure.

FGDG	Fraction	Aluminium	Copper	Iron	Manganese	Nickel	Zinc
FGDG1	F_acid/ exe_	30.87^a^ ± 2.2	nd	33.01^a^ ± 2.9	3.46^b^ ± 0.1	nd	1.08^ab^ ± 0.4
	F_red_	165.04^a^ ± 28.3	nd	70.37^a^ ± 13.7	0.99^a^ ± 0.1	0.35^a^ ± 0.1	2.21^a^ ± 0.2
	F_oxi_	80.22^b^ ± 6.6	nd	19.67^b^ ± 3.4	0.32^b^ ± 0.0	nd	0.55^b^ ± 0.1
	F_res_	1557.33^a^ ± 28.5	1.38 ± 0.5	2337.07 ± 8.8	69.99^a^ ± 7.9	6.58 ± 0.8	19.98 ± 4.6
Total		1833.46	1.38	2460.12	74.75	6.92	23.81
Bulk		1863.46^A^	1.51	2466.37	76.92	7.01	24.67
R_SCE_ (%)		98.39	91.41	99.95	97.18	98.75	96.52
FGDG2	F_acid/exe_	27.97^a^ ± 4.5	nd	32.44^a^ ± 3.2	4.91^a^ ± 0.2	nd	1.77^a^ ± 0.2
	F_red_	40.66^b^ ± 5.3	nd	24.67^b^ ± 12.3b	0.65^b^ ± 0.1	0.015^b^ ± 0.01	0.98^b^ ± 0.8
	F_oxi_	137.95^a^ ± 2.3	nd	69.80^a^ ± 7.9	0.62^a^ ± 0.1	nd	1.00^a^ ± 0.1
	F_res_	1619.23^a^ ± 92.5	1.40 ± 0.1	2569.85 ± 209.3	59.62^b^ ± 6.8	7.30 ± 1.3	19.98 ± 5.6
Total		1825.81	1.40	2696.77	65.81	7.32	23.73
Bulk		1827.26^A^	1.66	2697.10	68.18	7.00	24.93
R_SCE_ (%)		99.92	83.97	99.99	96.52	104.54	95.18
FGDG3	F_acid/exe_	11.53^b^ ± 1.2	nd	7.85^b^ ± 0.4	3.35^b^ ± 0.2	nd	0.72^b^ ± 0.2
	F_red_	56.99^b^ ± 29.5	nd	60.41^ab^ ± 18.8	1.19^a^ ± 0.1	0.13^b^ ± 0.03	1.48^ab^ ± 0.9
	F_oxi_	82.42^b^ ± 2.8	nd	25.20^b^ ± 1.3	0.40^b^ ± 0.01	nd	0.48^b^ ± 0.1
	F_res_	1359.90^b^ ± 75.5	1.16 ± 0.1	2356.99 ± 77.1	62.79^ab^ ± 8.0	6.91 ± 0.6	19.81 ± 1.3
Total		1510.85	1.16	2450.46	67.74	7.04	22.49
Bulk		1489.38^B^	1.42	2456.51	70.47	7.16	23.40
R_SCE_ (%)		101.44	81.26	99.75	96.11	98.37	96.11
Permissible limits of heavy metals in soil	Indian Std	NA	60–110	NA	NA	75–150	300–600
	EU	NA	50	NA	2000	75	300

nd, not detected; Values with different uppercase letters (A–B) in column are significantly different (*p* < 0.05) for bulk analysis of FGDG; values with different lowercase letters (a–b) in columns are significantly different (*p* < 0.05) for different fraction of FGDG samples. R_SCE_ (%), Recovery of sequential chemical extraction divided by bulk analysis results. Acid-soluble fraction (F_acid sol_), reducible fraction (F_red_), oxidizable fraction (F_oxi_), and residual fraction (F_res_).

As, B, Ba, Cd, Cr, Cu, Hg, Li, Mo, Pb, S, Sb, V were below the detectable limits.

Indian std: Indian standards Awashthi (2000).

EU, European Union Standards European Union (2006).

NA, Not available.

The presence of HMs in coal and lime are the source of metals present in the FGD gypsum and other by-products^33,32–35^. The coal samples are heterogenous in nature having varied compositions of elements^35^. Therefore, spatial and temporal study of coal material is important to estimate the variability in metal percentage in the different by-products of thermal power plants. Bhangare et al.^36^ studied the distribution of different trace elements in the coal and combustion residues (fly ash and bottom ash) from the five thermal power plants in India. The characterization of coal samples from the Vindhyachal thermal power plant is presented in supplementary Table S4. The furnace temperature contributes to the release of HMs during combustion (Attalla et al., 2004)^37^. The devices installed for pollution control in power plants and their operational environments also affect the partitioning of the HMs in different components of coal as well as gases (Hermine et al., 2012)^38^. The concentration of Al remained ~ 5200 mg kg^−1^ in the gas desulphurization system in NTPC when limestone was sprayed. Therefore, systematic investigation of used materials such as coal and limestone; operation of pollution control devices as well as combustion techniques will help in understanding the distribution of HMs in the FGD gypsums. The production of FGD gypsum is expected to be around 10–14 million metric tonnes per annum from 2024 to 2025 (https://cpcb.nic.in/uploads/hwmd/Guidelines_HW_5.pdf). Therefore, the huge production of FGD gypsums may be utilized as an alternative amendment to mineral gypsum for sodic land reclamation.

### Chemical speciation of metals

**Figure 4:**
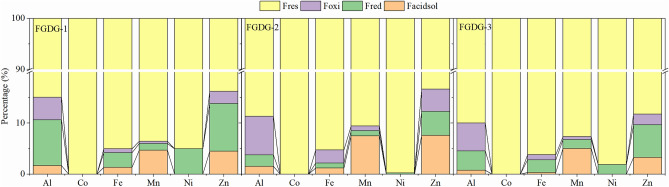
Chemical speciation percentage of heavy metals in the three FGD gypsums from selective sequential extraction procedure.

The estimation of total HMs in the by-products defines the level of contamination that affects the environment. However, the extent of the toxicity to the surrounding can only be expressed by the study of the behaviour of metal with respect to mobility, bioavailability, accumulation, or change from one form to another through a sequential extraction method. This methodology differentiates the metals into different behavioural groups viz*.,* the acid-soluble fraction (F_acid sol_), the reducible fraction (F_red_), the oxidizable fraction (F_oxi_), and the residual fraction (F_res_). The acid-soluble fraction is readily mobile and largely available to the environment, while F_red_ and F_oxi_ are only released under the presence of reduced/oxidized environment, and F_res_ form is considered the most stable form^21,39^. The sum of F_acid sol_, F_red_ and F_oxi_ refers to the mobile fraction. The HMs studied for the sequential extraction were Al, Co, Fe, Mn, Ni, and Zn for different FGD gypsums (Fig. 4). The reducible fraction (F_res_) contributed to almost 84–90% of total Al and this amount was consistent for all the collected FGD gypsums. Therefore, Al concentration was low in F_acid sol_, F_red_ and F_oxi_ fractions. A maximum percent of Cd resided in the residual phase. However, the release of Cd depends on soil reaction and the suitability of the environment^40^. The maximum portion of Ni was found as F_res_ phase, which accounted for more than 95% of total Ni. Therefore, a negligible amount of Ni stayed as F_red,_ and no Ni was found in the F_acid sol_ and F_oxi_ phases in FGD gypsum samples. Around 50–76% of Ni of the combustion mixture of coal resided in the residual phase^41,42^. However, Hao et al.^21^ reported 46.7–91.0% of the total Ni in the F_red_ and F_oxi_ phases. This variability may depend upon the natural mineralogy of the native coal and combustion technique adopted in different parts of the globe. The larger portion of Fe remained as the residual phase. The F_acid sol_, F_red_ and F_oxi_ fractions contained only 1–3% of total Fe. Similarly, about 90% of Mn and Zn remained in the residual form. In other phases, F_acid sol_, F_red_ and F_oxi_ carried around 7–16% of the total for both Mn and Zn. The association of Zn with Fe–Mn oxides of the combustion wastes has been recognized ^18^. Mn is believed to occur in carbonate and residual bound form extracted from coal or the limestone used in desulphurization process and transfer into the by-product in the form of gypsum^24,35,43^. The Co was not detected in F_acid sol_, F_red_ and F_oxi_ phases. It was only detected in the residual phase. Other researchers have reported the presence of Cd, Cr, Pb, As, and Cu in samples of FGD gypsum and fly ash from different power plants in China^18,21,24^. The study of heavy metals from 31 power plants in China reported cadmium content (0.01–2.10 mg kg^−1^) in the FGD gypsum samples higher than the soil quality standards of China^44^. Several other trace metals like Mo, Cr, Ni and Cd are also reported in a study carried out on reclamation of sodic soils through FGD gypsum application^45^.

The sequential extraction of FGD gypsum reported a larger concentration of HMs in the F_res_ phase (80–90%). The higher association with the residual fraction indicated its low bioavailability to biota. This observation supported the low risk of these metals to the environment. However, the speciation of metals present in FGD gypsum will depend upon the factors, such as combustion temperature, chemical characterization of flue gas, and operational parameters of the gas desulphurization process, causing various metal speciation and distribution in FGD gypsum from coal-fired power plants of different locations^21^.

### Leaching characteristics of HMs metals in the FGD gypsum

The organic and inorganic components present in the solid materials when exposed to the environment on weathering, rainfall, microbial action, or other related activities may cause environmental toxicity. The concentration of HMs in the deionized water/acid leachate from the three FGD gypsum samples collected from power plants is shown in Table 3. The different metals analyzed under leaching toxicity were Fe, Mn, Zn, Cd, Ni, Pb, As, Cr, Cu, and Mo. Among the different elements studied, only Mn, Ba and Cu were detected. Mn leached through the acid solution and was absent in the leachates of deionized water; while the leachates of the acid solution showed a negligible amount of Cu in FGD gypsum 1. Similarly, a negligible amount of Cu was detected in water soluble leachate of FGD gypsum 2. However, Cu was absent in leachates of FGD gypsum 3. Barium leached through both the SPLP solution/ deionized water. However, the concentration remained negligible according to the Toxicity Characteristic Leaching Procedure (TCLP) Regulatory Levels of the Resource Conservation and Recovery Act (EPA, 2014) (www.epa.gov). Researchers compared the leaching toxicity results with the standard limits of the Hazardous Waste-identification for extraction toxicity for the sewage leaching from the domestic waste landfills and the limit values of the leaching of inert waste landfills in European Community^28^.

**Table 3:** Leaching characteristics of heavy metals (mg kg^−1^) in different samples of FGD gypsum according to leaching tests.

FGDG	Extraction solution	Mn	Ba	Cu
FGDG1	Acid_sol_	1.89	2.56	0.05
	Water_sol_	nd	1.55	0.01
FGDG2	Acid_sol_	1.22	1.13	nd
	Water_sol_	nd	1.89	0.07
FGDG3	Acid_sol_	1.14	1.18	nd
	Water_sol_	nd	0.96	nd

nd, not detected; Fe, Zn, Cd, Ni, Cr were not detected in toxicity leaching test.

### Risk assessment code (RAC)

**Figure 5:**
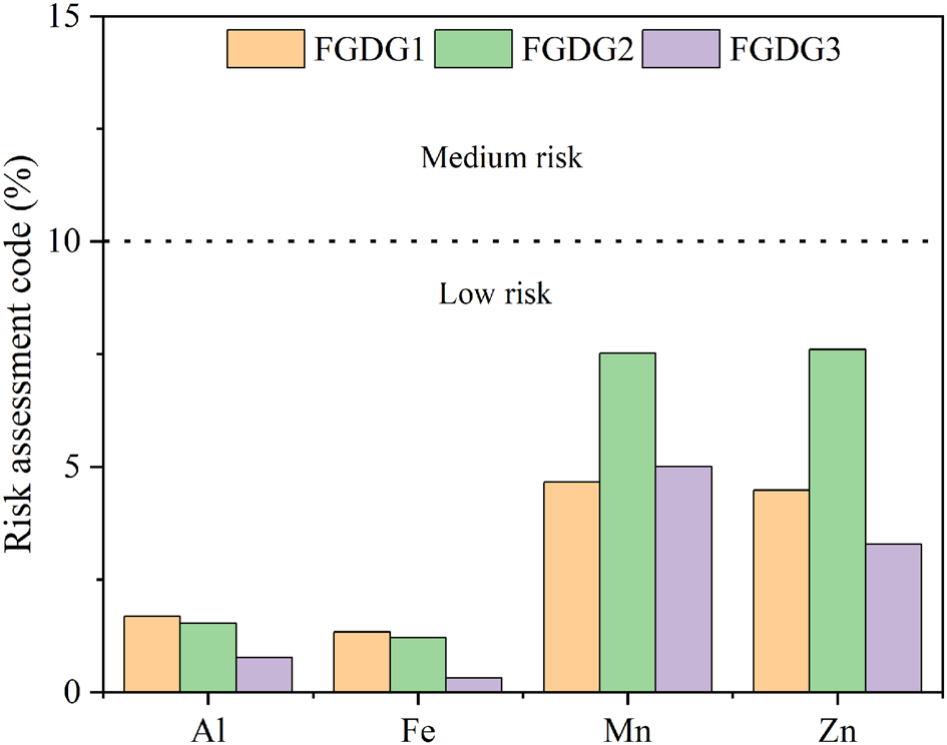
Risk assessment code of heavy metals in the heavy metals FGD gypsums.

The risk assessment code evaluates the movement of HMs from acid-soluble fraction (F_acid sol_) from FGD gypsum samples into the environment^36^. The results of RAC of different metals present in the FGD gypsum are displayed in Fig. 5 showing the level of risk to the environment. Neither Co and Ni were detected in the acid/water soluble fractions, nor had a risk to the environment. For Al, the three samples fell into the low-risk category. The risk levels of Mn, Fe, and Zn ranged from 4.6 to 7.9, 1.2 to 1.3, and 3.2 to 7.5. The RAC analysis of all the metals showed a low level of eco-toxicity. Therefore, the results depicted that the FGD gypsum will not pose any significant harmful effects on the health of the organisms in the ecosystem.

### Pollution indices for environmental risk assessment

The toxicity of trace metals from HMs accumulation in FGD gypsum differs from the total estimation as it is dependent on its availability, mobility, and transformation subjected to environmental conditions. FGD gypsum is an emerging amendment source for sodic soil reclamation showing negligible levels of risk and concern for the environment^32,46^. However, it is necessary to assess the soil contamination level as well as the ecotoxicological impacts of FGD gypsum when applied to soil for sodic soil reclamation. Nevertheless, the application of FGD gypsum depends upon the presence of alkalinity (CO_3_^2−^/HCO_3_^−^) and degree of soil sodicity *i.e*. the presence of Na^+^ in soil solution and exchange phase^47,48^. The standard rate of application of gypsum for sodic soil reclamation is 10 tonnes per hectare^49^. Therefore, the chances of possible contamination of soil through the prescribed application rate of FGD gypsum for the reclamation of sodic soils were estimated to extrapolate the extent of risk to soil system. The contamination factor calculated for metals present in different FGD gypsum samples showed no contamination (C_f_ 0.0–0.2) transfer in the soil through the application of FGD gypsum @ 10 t ha^−1^(Table 4). The enrichment factor is another index used to assess the toxicity of metals in the soil. The enrichment of different metals in FGD gypsum remained in order: Al < Mn < Co < Zn < Ni. Metals such as Ni (E_f_ 4.3–5.2), Zn (E_f_ 4.5–5.6), Mn (E_f_ 1.2–2.0), and Co (E_f_ 1.7–2.8) were below the national and internal standard limits^50,51^ and it will cause low enrichment of metals into the soil upon application (Table 5). However, the sodic soils are reported to be deficient in Zn^52^. The enrichment of Zn will help in the Zn-fertilization of the soil. The geo-accumulation values (I_geo_ < 1) of FGD gypsum reported that its application FGD gypsum in sodicity reclamation would not add any toxic level concentration of heavy metals to soil (Table 3).

**Table 4:** Contamination factor (C_f_) and geo-accumulation index (I_geo_) values of elements present in the FGD gypsum samples.

FGDG	Al	Fe	Mn	Ni	Zn	Co
Contamination factor (C_f_)						
FGDG1	0.0001	0.0003	0.0006	0.0016	0.3475	0.1513
FGDG2	0.0001	0.0003	0.0005	0.0016	0.3511	0.1663
FGDG3	0.0001	0.0003	0.0005	0.0016	0.3296	0.1423
Geoaccumulation index (I_geo_)						
FGDG1	–13.825	–12.219	–11.361	–9.907	–9.925	–11.149
FGDG2	–13.853	–12.092	–11.534	–9.909	–9.922	–10.981
FGDG3	–14.147	–12.225	–11.489	–9.875	–10.007	–11.213

**Table 5:** Enrichment factor (E_f_) values of elements present in the FGD gypsum samples.

FGDG	Al	Mn	Ni	Zn	Co
FGDG1	0.329	1.819	4.972	4.928	2.144
FGDG2	0.296	1.485	4.549	4.534	2.162
FGDG3	0.264	1.677	5.101	4.706	2.033

### Changes in soil pH_s_, EC_e,_ and SAR_e_ after incubation

**Figure 6:**
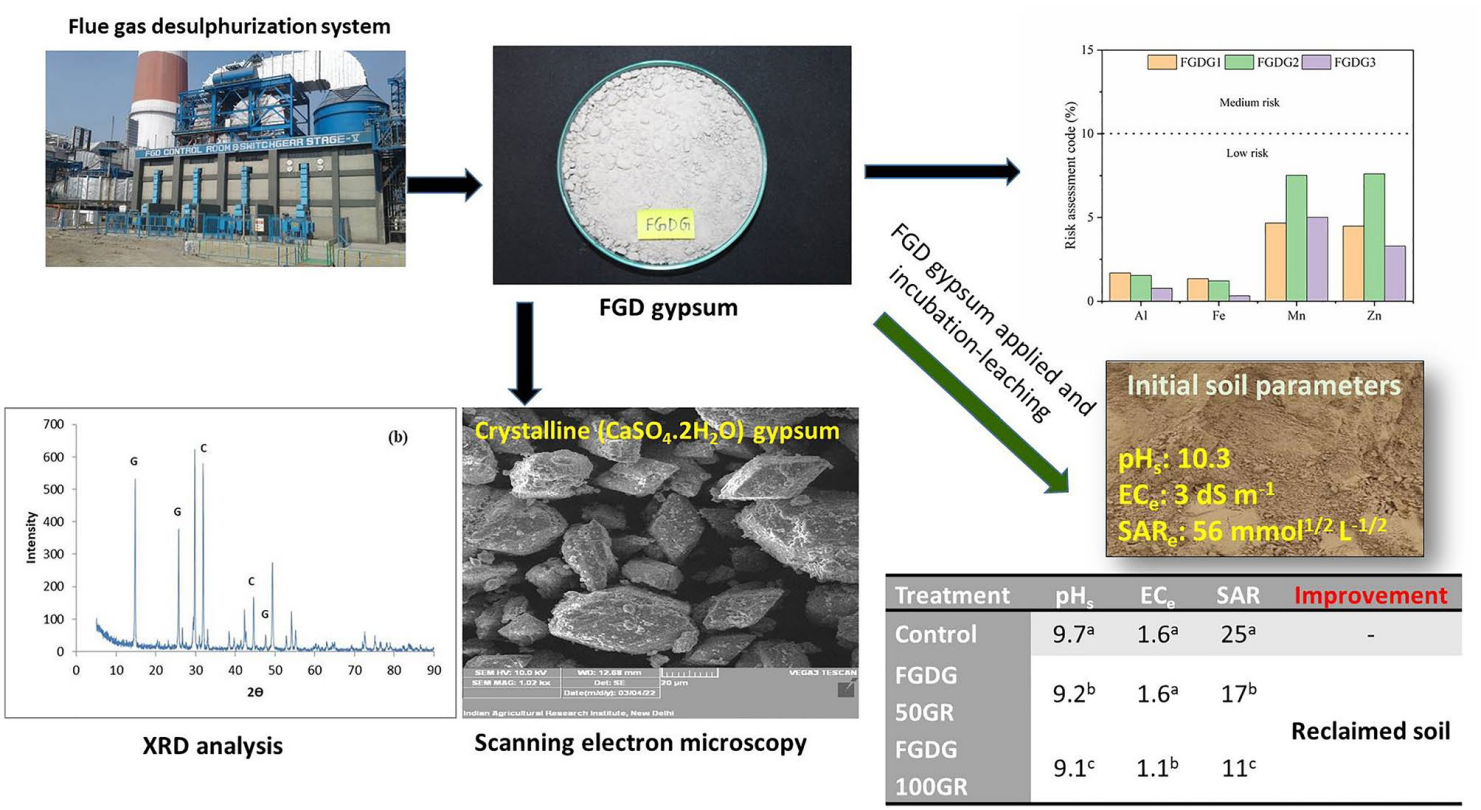
Utilization of FGD gypsum in reclamation of sodic soil.

Amending soil with FGD gypsum (50GR and 100 GR) significantly decreased the soil pH_s_ (pH of soil water saturation paste) up to 1.09–1.22 (*P* > 0.05). The EC_e_ (electrical conductivity of soil water saturation paste extract) was reduced by 1.35–1.92 units in unamended and treated soils (Table 6). The SAR_e_ (SAR of soil water saturation paste extract) of the soil was significantly reduced with the application of FGD gypsum. There was 26 percent decrease in the total alkalinity of the soil with the application of 100GR FGD gypsum compared to the unamended soil (Fig. 6). The soil reclamation with the application of FGD gypsum showed a significant reduction in pH and water-soluble Na^+^, Cl^−^, and CO_3_^2−^ + HCO_3_^−^ of the sodic soils in China^41,53,54^.

**Table 6:** Improvement in soil properties on application of FGD gypsum and leaching in sodic soil.

Treatment	pH_1:2_	EC_1:2_ (dS m^−1^)	pH_s_	pH_e_	EC_e_ dS m^−1^	Total Alkalinity (mmoles L^−1^)	Sodium absorption ratio (mmol^1/2^ L^−1/2^)	CaCO_3_ (%)
Control	10.23^a^	1.25^a^	9.70^a^	9.17^a^	1.64^a^	9.13^a^	25.05^a^	1.13^a^
FGDG 50GR	9.85^b^	1.10^b^	9.22^b^	8.03^b^	1.62^a^	6.44^a^	16.67^b^	0.42^c^
FGDG 100GR	9.61^c^	0.76^c^	9.09^c^	8.37^b^	1.07^b^	6.75^a^	11.18^c^	0.64^b^
LSD	0.03	0.05	0.13	0.36	0.27	5.29	2.11	0.09

LSD, Least significant difference; values with different lowercase letters (a–b) in columns are significantly different (*p* < 0.05).

## Conclusion

The FGD gypsum, a by-product of the coal industry was characterized and evaluated for heavy metal toxicity to use as a futuristic alternative amendment for sodic soil reclamation. The XRD, SEM, and elemental characterization confirmed the presence of the crystalline (CaSO_4_·2H_2_O) gypsum as the dominant mineral present in the FGD gypsum by-product, other impurities like Si and Mg corresponded to the presence of quartz and calcite. The presence of calcium in the FGD gypsum significantly reduced the pH_s_ and SAR_s_ of the sodic soil. The total heavy metal concentration followed the order of Fe > Al > Mn > Zn > Ni > Co. The maximum percentage of metals studied under sequential extraction remained in a more stable form (F_res_ phase ~ 80–90%) which are considered hard to release unless in adverse weathering conditions. Leaching toxicity showed no toxicity of metals while, RAC analysis showed a low level of eco-toxicity of Mn, Fe, and Zn. The results of the environmental indices further ascertained no contamination (C_f_ 0.0–0.16) of FGD gypsum to the environment. However, Mn and Co showed minor enrichment and Ni and Zn showed moderate enrichment in the soil which might be good for improving the micronutrient concentration in deficient sodic soil. The low geo-accumulation values (I_geo_ < 1) of FGD gypsum indicated no addition of any toxic metal to the soil upon application FGD gypsum for soil reclamation and thereby, transferred to humans through edible crops. This study revealed the possibility of FGD gypsum as a safe and environmentally sustainable alternative amendment for the reclamation of sodic soil.

## Materials and methods

### Sample collection from the power plant

The FGD gypsum as by-product of the FGD system was received from the wet FGD system of the coal power plant of NTPC, Vindhyachal, Singrauli, Madhya Pradesh, India. The FGD gypsum samples were collected at different time intervals. The first sample was collected in May 2020 (FGD gypsum 1), the second sample (FGD gypsum 2) was collected in August 2020, third sample (FGD gypsum 3) in June 2021. The three samples as received (without purification) were further used for mineral, elemental, and heavy metals characterization and other experimental works.

### Sample characterization

#### Physico-chemical analysis

The pH and EC of FGD gypsum were measured in a 1:2 material-water suspension using a glass electrode and conductivity meter, respectively*.* The CaCO_3_ percentage was calculated following the manometric method using Collin's calcimeter method of Allison and Moodie^55^. For moisture content estimation, the FGD gypsum samples were weighed and dried in a hot air oven at 105 °C for 48 h, and volumetric gypsum moisture content was expressed as percent weight loss on a volume basis (Table 1).

#### X-ray diffraction analysis

X-ray diffraction analysis of the powdered FGD gypsum samples was performed using Phillips diffractometer with Ni-filtered Cu Kα (λ = 1.5418 Å) source operating at 40 kV and 20 mA. The diffraction pattern was recorded at a scanning speed of 2°2θ min^−1^ in the 2θ range between 5° and 90°.

#### Scanning electron microscopy

A VEGA3 LM scanning electron microscope (SEM) (Tescan Orsay Holding Instrument, Czech Republic) having backscattered electron (BSE) and secondary electron (SE) detectors were used to acquire the SEM images of the FGD gypsum samples to analyze the surface morphology.

### Analysis of trace elements

#### Bulk analysis

The FGD gypsum samples collected from the NTPC unit were grounded and sieved with a 2 mm sieve for bulk analysis. Approximately 0.5 g of the sample was digested adding 10 mL of concentrated HNO_3_, 5 mL HClO_4_, and 10 mL HF acid at 135 °C. The digestion process was repeated with an acid mixture till the dissolution of the FGD gypsum samples^21^. The solution was filtered through Whatmann no. 42 after the process completion and diluted to a standard volume of 50 mL with distilled water. The elemental composition of FGD gypsum was carried out using Inductively Coupled Plasma Emission Spectroscopy (ICP-OES) (ICPE-9000, Shimadzu, Japan). Meanwhile, the precision of the process was ensured by analysis of HMs in the certified material, *i.e*., Periodic Table Mix 1 (ISO/IEC 17025 & ISO 17034), and blanks.

#### Sequential chemical extraction

The chemical speciation of the trace elements was done by the selective sequential extraction (SSE) procedure described by Rauret et al.^36^. This process categorizes the sample components into different behavioural classes. The description of the extraction procedure is displayed in Fig. 1. The extracted fractions (leachate) collected from each step were centrifuged at 3000 rpm for 20 min. and the supernatant separated was filtered with a 0.45 μm cellulose acetate membrane filter, and stored at 4 °C before determination of elemental concentration by ICP-OES.

### Leaching toxicity

The leaching test of the HMs present in FGD gypsum samples was done following the US EPA SPLP standard to extract the acid-soluble fraction (USEPA, Method 1312, 1994) and European Standard leaching test EN 12457-2 (2002) for water-soluble fraction. The extraction fluid was prepared by mixing concentrated sulfuric acid with nitric acid (mass ratio 2:1) with the pH value adjusted to 3.20 ± 0.05. The solution-to-sample ratio taken was 10:1. Samples were extracted by end-over-end tumbling at 30 rpm for 18 ± 2 h at room temperature followed by centrifugation for 20 min at 3000 rpm. The supernatant was filtered through a 0.45 μm cellulose acetate membrane filter for further analysis by ICP-OES. The water-soluble HMs in FGD gypsum were analyzed by leaching the samples for 24 h with ultra-pure water (Liquid to solid ratio = 10:1) generated through PureLab Classic ELGA (UK). The leachate separation was done by vacuum filtration with a 0.45 μm filter paper before the determination of HMs.

### Quality control

The total concentration of HMs in the FGD gypsum samples (dry weight basis) was estimated in triplicates. The mean (± standard deviation) following the standard addition method ensures the accuracy of estimation for the standard recovery rate calculations described by Hao et al.^21^. The R_SCE_ (%) defines the recovery of sequential chemical extraction which was calculated by the data of the sum of four forms divided by bulk analysis result. The recovery rates ranged from 81 to 105%. The sample analysis included the blank samples to avoid human error if any.

### Risk assessment code (RAC)

Risk assessment code addresses the extent of eco-risk caused by the labile fraction of the HMs present in the samples. RAC categorizes the risk level into five classes. Class I as no risk when HMs in the mobile fraction (F1 fraction) are > 1% of the total concentration, 1–10% is low risk, 11 to 30% is medium risk, 31–50% is high risk, and > 50% is very high risk (Yan et al. 2010)^56^.

### Pollution indices

#### Contamination factor (C_f_)

The C_f_ evaluated the pollution level associated with the single element using Eq. (1)^57,58^.

1 $$Cf_{x} = \frac{{\left[ X \right]_{FGDG} }}{{\left[ X \right]_{crust} }}$$

where [X_FGD gypsum_] and [X_crust_] are the concentration of the element in the FGD gypsum and earth crust respectively. The categorization of the contamination factor is given in Supplementary Table S1.

#### Enrichment factor (E_f_)

The enrichment factor was calculated with reference to the concentration of Fe used for geochemical normalization^35,59,60^ using Eq. (2)^61,62^.

2 $$Ef = \frac{{(C_{i} /C_{Fe)FGDG} }}{{(C_{i} /C_{Fe)reference} }}$$

where C_i_ and C_Fe_ is the concentration of an element and Fe in each FGD gypsum sample and reference soils, respectively. The categorization of the enrichment factor is given in Supplementary Table S2.

#### Geoaccumulation index (I_geo_)

Geoaccumulation index was calculated *wrt* the earth’s crust concentration using Eq. (3)^63^.

3 $$I_{geo} = log_{2} \left[ {\frac{Cn}{{1.5 Bn}}} \right]$$

where Cn and Bn are concentration of elements in the FGD gypsum and earth crust respectively; factor 1.5 minimizes the lithogenic variations in the soil. Different categories of I_geo_ are given in Supplementary Table S3.

### FGD gypsum-based soil sodicity reclamation study

The gypsum requirement of bulk soil collected from Belau, Azamgarh, Uttar Pradesh, India (Latitude 25° 56.350′ Longitude 82°57.099′) for complete neutralization (G100) of sodicity was determined with a value of 7.6 Mg ha^−1^ ^64^. Then triplicated set of soil was incubated with respective doses viz., control, FGD gypsum @ 50% recommended doses of mineral gypsum (FGD gypsum 50 GR) (T2), FGD gypsum @ 100% recommended doses of mineral gypsum (FGD gypsum 100 GR) at 60% water holding capacity at room temperature (27–32 °C). Soils were leached with de-ionzed water at 30 and 60 days after incubation with two pore volumes of the water. After 60 days of incubation (DAI), the soil samples were drawn and used for the analysis of solid and solution phase parameters. The samples were air-dried and ground in wooden pestle mortar and passed through a 2-mm mesh sieve. pH and EC of soil were measured in a 1:2 soil–water suspension using a glass electrode and conductivity meter, respectively^65^. The soil pH_s_ and EC_e_ were determined by measuring the pH of the soil water saturation paste and the electrical conductance of the soil water saturation paste extract with a conductivity meter^66^. Calcium carbonate equivalent was measured by neutralization with HCl^52^. Ca^2+^ and Mg^2+^ were measured by AAS (Analytika Jena, ZEEnit 700p; Germany). A flame photometer was used for the determination of Na^+^ and K^+^ (Systronics, India). Total alkalinity were determined by methyl red, phenolphthalein, and bromocresol green endpoint titration, respectively^67^. Nephelometer (SI98713; Hanna, Romania) was used for the determination of $({\text{SO}}_{4}^{2 - } )$^68^. Sodium absorption ratio (SAR) is the mathematical relationship with the ions as shown in the following equation:

$$SAR = \frac{{[{\text{Na}}^{ + } ]}}{{\sqrt {[{\text{Ca}}^{2 + } + {\text{Mg}}^{2 + } ]/2} }}$$

### Statistical analysis

Data generated from the experiments were analyzed with SAS 9.3. The Kruskal–Wallis test was performed for the analysis of variance. All pair-wise comparisons were made using *p* values (*p* < 0.05) adjusted by the Bonferroni correction for multiple tests.

### Ethical approval

Ethical approval (Research article 25/2023, dated 15.05.2023) was obtained from the Project Monitoring and Evaluation Cell headed by the Director, CSSRI, Karnal (India).

### Supplementary Information

Supplementary Tables.

## Data Availability

Data available within the article and supplementary materials.
