# Supplementary material for: Characterization of flue gas desulphurized (FGD) gypsum of a coal-fired plant and its relevant risk of associated potential toxic elements in sodic soil reclamation
Source: Sci Rep. 2023 Nov 13;13:19787. doi: 10.1038/s41598-023-45706-y (PMC10643664; doi:10.1038/s41598-023-45706-y)
Supplement: Supplementary file 1 — Supplementary Tables. [file 41598_2023_45706_MOESM1_ESM.docx]

**Title: Characterization of flue gas desulphurized (FGD) gypsum of a coal–fired plant and its relevant risk of associated potential toxic elements in sodic soil reclamation**

**Running Title:** **Characterization and Risk Assessment of Flue Gas Desulphurization (FGD) Gypsum**

### Authorline: Parul Sundha^1#^, Raj Mukhopadhyay^1#^, Nirmalendu Basak^1#^*, Arvind Kumar Rai^1#^*, Sandeep Bedwal^1^, Subedar Patel^1^, Sanjay Kumar^1^, Harshpreet Kaur^1^, Priyanka Chandra^1^*, Parbodh Chander Sharma^1^, Sanjeev Kumar Saxena^2^, Somendra Singh Parihar^2^ and Rajender Kumar Yadav^1^

^1^ICAR–Central Soil Salinity Research Institute, Karnal 132 001, Haryana, India

^2^National Thermal Power Corporation, Vindhyachal, Singrauli, Madhya Pradesh, India

^#^These authors contributed equally to this work

*Corresponding authors email: [nirmalendubasak@rediffmail.com](mailto:nirmalendubasak@rediffmail.com); nirmalendu.basak@icar.gov.in (N Basak; orcid.org/0000-0002-2281-0253) ak.rai@icar.gov.in (AK Rai; orcid.org/0000-0002-8045-9828); [priyanka.chandra@icar.gov.in](mailto:priyanka.chandra@icar.gov.in)

Telephone and Fax no.: +91-9416145187; +91-8814878225; +91–8295406495;

+91-0184-2290-480

**Table Supplementary S1**. Categories of contamination factor (C_f_ )

| C_f_ value | Contamination |
| --- | --- |
| < 1 | low |
| 1-3 | moderate |
| 3-6 | considerable |
| > 6 | very high |

**Table Supplementary S2**. Categories of enrichment factor (E_f_)

| E_f_ Value | Enrichment of soil |
| --- | --- |
| < 1 | no enrichment |
| 1<3 | minor |
| 3–5 | moderate |
| 5–10 | moderate-to-severe |
| 10–25 | severe |
| 26–50 | very severe |
| >50 | extremely severe |

**Table Supplementary S3.** Contamination status according to geoaccumulation index (I_geo_) values

| Values of I_geo_ | sludge contamination status |
| --- | --- |
| ≤ 0 | uncontaminated |
| 0< I_geo_≤1 | uncontaminated to moderate |
| 1 < I_geo_≤ 2 | moderate |
| 2 < I_geo_≤3 | moderate to high |
| 3 < I_geo_≤ 4 | high to extreme |
| > 5 | highly to extremely high polluted |
| 5-6 | extreme |

Table S4: Average elemental concentrations (μg/g) in the feed coal of power plant (Adopted from Bhangare et al., 2011^1^)

| Vindhyachal thermal power plant, Madhya Pradesh | Cd | Ni | Li | Cr | Co | Mn | Mg | Pb | Zn | Cu | Fe | As | Hg |
| --- | --- | --- | --- | --- | --- | --- | --- | --- | --- | --- | --- | --- | --- |
|  | 0.31 | 15.43 | 1.90 | 13.29 | 4.33 | 148.51 | 6.49 | 3.25 | 160.11 | 13.42 | 14250 | 1.55 | 4.40 |

1. Bhangare, R. C., Ajmal, P. Y., Sahu, S. K., Pandit, G. G. & Puranik, V. D. Distribution of trace elements in coal and combustion residues from five thermal power plants in India. *International Journal of Coal Geology* **86**, 349–356 (2011).
